# First-Principles Investigation of Ag Doping Effects on Phase Stability and Mechanical Properties in Rare-Earth Magnesium Alloy Mg_24_(Gd,Y)_5_

**DOI:** 10.3390/ma19040797

**Published:** 2026-02-18

**Authors:** Jiachun Yuan, Dengjun Wu, Jiamin Li, Juan Hou, Hao Wang

**Affiliations:** 1 School of Materials and Chemistry, University of Shanghai for Science and Technology, Shanghai 200093, China; yuanjchun@163.com (J.Y.); djwu0930@163.com (D.W.); 2 School of Materials Science and Engineering, South China University of Technology, Guangzhou 510006, China; li_jiamin0521@163.com; 3 Institute of Metal Research, Chinese Academy of Sciences, Shenyang 110016, China

**Keywords:** first-principles calculations, Mg_24_(Gd,Y)_5_, Ag atom, crystal structure

## Abstract

**Highlights:**

**What are the main findings?**

- Ag preferentially occupies Mg sites in Mg_24_(Gd,Y)_5_ and segregates in rare-earth-enriched regions, validated by experiments.
- Ag forms covalent-ionic bonds with RE atoms via orbital hybridization, enhancing phase stability.
- Ag doping increases alloy ductility (fracture strain from 4% to 12%) with moderate UTS reduction, optimizing strength-plasticity synergy.
- First-principles calculations combined with WAAM experiments establish a micro-macro property correlation.

**What are the implications of the main findings?**

- Clarifies the atomic-scale mechanism of Ag doping in Mg-Gd-Y alloys, filling the gap in existing studies.
- Provides a novel strategy to improve ductility of high-strength rare-earth Mg alloys without severe strength loss.
- Offers theoretical and experimental basis for compositional design of Mg-Gd-Y-Ag-Zr alloys for additive manufacturing.
- Validates the effectiveness of integrating DFT calculations and WAAM technology in alloy performance optimization.

**Abstract:**

The limited ductility of the VW63K rare-earth magnesium alloy fabricated via cold metal transfer wire arc additive manufacturing (CMT-WAAM) was targeted in this work. An integrated approach that combines first-principles calculations with experimental characterization was employed to achieve this goal. This approach was used to systematically investigate how Ag doping alters the microstructure and mechanical properties of the alloy. First-principles calculations performed on the primary precipitate phase Mg_24_(Gd,Y)_5_ demonstrated that Ag atoms preferentially occupy the Mg lattice sites and form pronounced orbital hybridization with neighboring rare-earth atoms. These interactions were found to enhance critical mechanical parameters, including the Cauchy pressure, B/G ratio, and Poisson’s ratio, which are indicative of enhanced ductility and toughness of the phase. Experimental results indicate that the fracture strain of the VW63K-Ag alloy was increased from approximately 4% to above 12% following Ag doping. This resulted in a significant improvement in ductility. The ultimate tensile strength (UTS) underwent only a moderate reduction. Using a closed-loop approach integrating theoretical prediction and experimental validation, the microstructural modification and strengthening mechanisms of Ag in the VW63K alloy fabricated via CMT-WAAM were clarified. These findings offer a theoretical foundation and experimental evidence for compositional design and optimizing additive manufacturing (AM) processes for rare-earth magnesium alloys.

## 1. Introduction

Magnesium and its alloys represent an important class of lightweight structural materials due to their low density, high specific strength, and excellent recyclability. This combination of properties has facilitated their broad application in the automotive, aerospace, and consumer electronics industries [1]. However, conventional magnesium alloys still suffer from notable limitations, including low strength and limited ductility, poor creep and wear resistance, and high corrosion rates [2]. These issues are exacerbated by traditional casting processes, which typically introduce defects such as porosity and inclusions as well as a coarse-grained microstructure. Such processing-induced flaws ultimately limit further improvements in mechanical properties in the cast components [3,4]. With the rapid development of additive manufacturing technologies, Wire Arc Additive Manufacturing (WAAM) has emerged as a promising technique for fabricating high-performance, complex magnesium alloy components [5,6,7]. Studies have shown that the high cooling rates inherent to WAAM promote the rapid solidification of the molten pool. Consequently, grain refinement and microstructural homogenization can be achieved. For instance, the Mg–6.5Al–0.8Zn–0.3Mn alloy fabricated via WAAM was found to exhibit a uniform equiaxed microstructure with an average grain size of 27 μm, which resulted in high hardness and enhanced mechanical properties [8]. Ying et al. [9] utilized WAAM to fabricate the AZ61 magnesium alloy, which yielded a fine equiaxed microstructure with an average grain size of 31.6 μm and a density of 99.669%. The resulting specimens exhibited an elongation to fracture of 14% and an ultimate tensile strength (UTS) of 247 MPa. They emphasized that the coordinated control of key process parameters—including current, voltage, and wire feed speed—dictates the final grain size and relative density of the microstructure. This relationship provides a critical foundation for optimizing the mechanical properties of WAAM-fabricated magnesium alloys.

In recent years, rare-earth magnesium alloys have emerged as a key research area in materials science, primarily due to their superior mechanical properties [10]. The addition of rare earth elements to Mg-Gd-Y-Zr alloys significantly enhances their properties. Synergistic solid-solution strengthening, grain refinement, and age hardening further improve these alloys’ comprehensive properties [11,12]. Notably, tuning rare earth concentrations enables precise alloy design and mechanical performance optimization. Nodooshan et al. [13] tuned the Gd concentration (x = 3, 6, 10, 12 wt.%) in Mg–xGd–Y–Zr alloys and observed that the age-hardening response and tensile strength were markedly increased with increasing Gd content. They also successfully fabricated an Mg-10Gd-3Y-0.5Zr alloy exhibiting a tensile strength of 390 MPa and a yield strength of 245 MPa. Wang et al. [14] examined the effect of Y content on the properties of Mg-10Gd-xY-0.4Zr alloys, where x = 1, 3, and 5 wt.%. Their findings showed that increasing the Y content was found to enhance both the age-hardening response and tensile properties. This enhancement was most significant at 5 wt.% Y, where the alloy exhibited an ultimate tensile strength (UTS) of 302 MPa and a yield strength (YS) of 289 MPa. Another study demonstrated that the Mg-10Gd-2Y-0.5Zr (wt.%) alloy was found to exhibit superior overall mechanical performance [15], exhibiting an ultimate tensile strength (UTS) of 403 MPa, a yield strength (YS) of 311 MPa, and an elongation of 15.3%. However, Jiang et al. [16] observed that increasing rare-earth concentrations was found to induce the formation of additional precipitates. This significant strength improvement from enhanced precipitation is often accompanied by a significant reduction in ductility. As a result, the practical application of high-strength rare-earth magnesium alloys is still limited by their inherent limited ductility.

The addition of alloying elements (e.g., Zn, Al, Ag) is an effective strategy for modulating microstructure and synergistically optimizing strength–toughness properties [17,18]. Specifically, the addition of Zn and Al has been shown to lead to simultaneous strength enhancement and ductility improvement, which is attributed to mechanisms such as grain refinement and modification of precipitate morphology and distribution. Ag, due to its unique atomic size and electronic structure, has shown exceptional promise for tailoring comprehensive properties of Mg-Gd-Y alloys. Huang et al. [19] demonstrated that, in the Mg-8.9Gd-1.8Y-0.5Zr-0.2Ag (wt.%) alloy, the combined effects of precipitation strengthening and enhanced ductility from grain-boundary precipitate-free zones were found to produce a good balance of strength and ductility following aging at 498 K. Yamada et al. [20] reported that aging treatment was found to increase the alloy’s ultimate tensile strength (UTS) to 410 MPa, which stemmed largely from the synergistic strengthening effect of basal precipitates and the prismatic β′ phase. Zhang et al. [21] further demonstrated that the addition of trace Ag to the as-cast T6 Mg-7.5Gd-1.5Y-0.4Zr alloy significantly enhances the alloy’s ultimate tensile strength (UTS) beyond 400 MPa. This strength improvement was primarily ascribed to an increased volume fraction of β′ phase and the formation of basal disk-like precipitates. Beyond the fundamental strengthening and toughening mechanisms, Ag-doped Mg-Gd-Y-Zr alloys hold significant promise for advanced technological applications. The improved strength-ductility synergy, coupled with the inherent lightweight nature of magnesium alloys, makes them strong candidates for weight-sensitive structural components, such as missile body casings and control fins in military applications [22].

While numerous studies have been reported on the improved mechanical properties of magnesium alloys through Ag alloying, investigations into the stability of Ag in these alloys and the underlying physical mechanisms remain scarce. Compared with experimental approaches, first-principles calculations based on density functional theory (DFT) provide a distinct advantage, which enables the direct simulation of material properties and electronic structures at the atomic and electronic levels. In this work, the first-principles calculations based on DFT were employed. The site preference and electronic structure features of Ag atoms in the key precipitate phase Mg_24_(Gd,Y)_5_ in Mg-Gd-Y-Zr-based alloys were systematically investigated. Their effects on the phase stability and chemical bonding of this precipitate were also explored. Furthermore, the effect of Ag doping on regulating the alloy’s macroscopic stiffness, hardness, and toughness was evaluated through elastic constant calculations. The underlying micro-mechanism of Ag doping was further uncovered at the atomic scale. To verify theoretical predictions and facilitate practical applications, the Ag-microalloyed Mg-Gd-Y-Zr alloy specimens were fabricated using WAAM. The microstructure, phase constitution, and mechanical properties of these specimens were systematically characterized using standard experimental techniques. Excellent consistency was observed between the theoretical simulations and the experimental results. This work provides a systematic theoretical basis and technical support for the composition design, processing optimization, and development of high-performance Mg-Gd-Y-Ag-Zr alloys.

## 2. Computational Models and Methods

### 2.1. Computational Models

**Figure 1:**
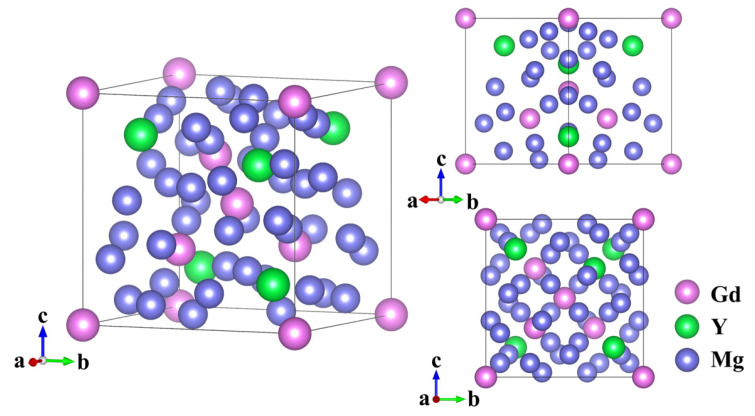
Crystal structure of Mg_24_(Gd,Y)_5_.

Figure 1 presents the crystal structure of the Mg_24_(Gd,Y)_5_ phase. This alloy phase is classified as a cubic crystal system, with its unit cell exhibiting a body-centered cubic (BCC) structure and a space group of I43m. The structural model was constructed using Mg_24_Y_5_ as the parent crystal structure. This initial model was composed of 48 Mg atoms and 10 Y atoms. These atoms were located at specific Wyckoff positions consistent with the crystallographic symmetry: 24g (Mg), 24g (Mg), 2a (Y), and 8c (Y) [23]. From this initial configuration, the five Y atoms were replaced with Gd atoms. Specifically, Gd atoms occupy the 2a Wyckoff sites (2 atoms) and partially substitute Y at the 8c sites (3 atoms). In comparison, the remaining 5 Y atoms retain their positions at the 8c sites, thereby forming the structural model of Mg_24_(Gd,Y)_5_.

**Figure 2:**
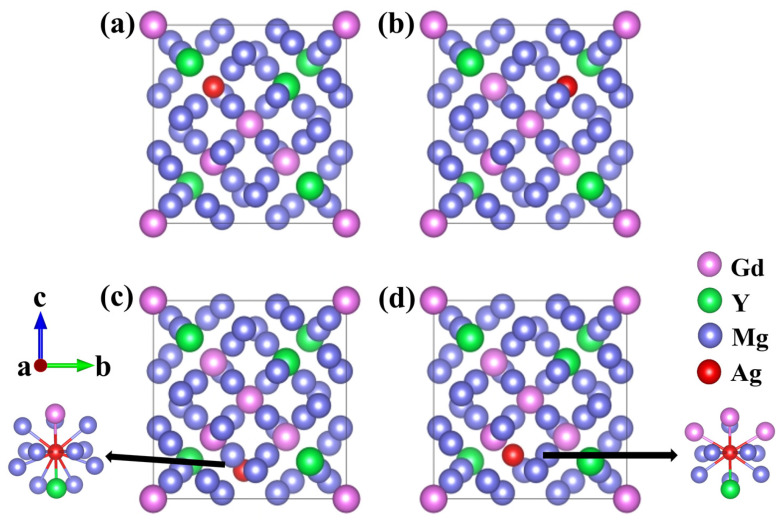
Schematic diagrams of unit cells: (**a**) Mg_48_Gd_4_Y_5_Ag; (**b**) Mg_48_Gd_5_Y_4_Ag; (**c**) Mg_47_Gd_5_Y_5_Ag-1; (**d**) Mg_47_Gd_5_Y_5_Ag-2.

Existing research has shown that Ag atoms are preferentially incorporated into the Mg-Gd-Y-Zr alloy lattice through substitution rather than being incorporated into interstitial sites [24]. Accordingly, the present work focuses exclusively on substitutional Ag doping. Based on the Wyckoff site distribution, two types of Mg sites exist in Mg_24_(Gd,Y)_5_: these sites correspond to the same 24g Wyckoff position but exhibit distinct coordination environments, with details summarized in Table 1. To systematically examine the site preference of Ag atoms, four distinct substitutional solid-solution models were constructed. In these models, a single Ag atom replaced either one Gd, one Y, or one Mg atom. The corresponding models are Mg_48_Gd_4_Y_5_Ag, Mg_48_Gd_5_Y_4_Ag, Mg_47_Gd_5_Y_5_Ag-1, and Mg_47_Gd_5_Y_5_Ag-2, as illustrated in Figure 2.

### 2.2. Calculation Methods

All calculations were performed using density functional theory (DFT), as implemented within the Vienna ab initio Simulation Package (VASP.5.4). The exchange-correlation interactions were described using the Perdew-Burke-Ernzerhof (PBE) functional under the generalized gradient approximation (GGA) [25]. All electron-ion interactions were represented by the projector augmented wave (PAW) method [26], using standard potentials from the VASP library [27]: Mg (Mg_sv, 3s^2^), Gd (Gd, 4f^7^5d^1^6s^2^, with 4f^7^ treated as core electrons), Y (Y, 4d^1^5s^2^), and Ag (Ag, 4d^10^5s^1^). No Hubbard U correction was applied in this study. For Brillouin zone integration, the Monkhorst-Pack method was utilized to generate k-point meshes [28]. The convergence thresholds for energy and force were defined as 1 × 10^−5^ eV per atom and 0.02 eV/Å, respectively.

**Figure 3:**
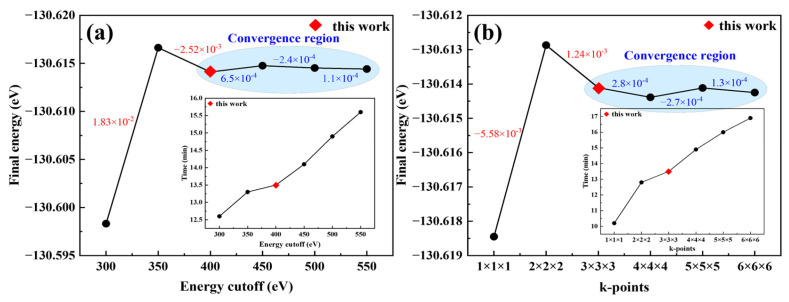
Convergence test results of the calculation parameters: (**a**) Energy cutoff; (**b**) k-point mesh.

For the energy cutoff (Ecut) and k-point mesh, larger values extend the relaxation time, while smaller ones compromise accuracy. Convergence tests were thus performed to balance computational speed and precision. Parameters were considered acceptable when the resulting energy variation remained within 10^−3^ eV. Convergence test results for Ecut and the k-point mesh are presented in Figure 3, respectively; the values labeled in the figures correspond to the energy differences between consecutive data points. From these results, when Ecut was set to 400 eV, the energy variation from structural optimization approached the 10^−3^ eV convergence criterion, achieving the required computational precision. Meanwhile, the k-point mesh was found to meet the energy convergence criterion at a 3 × 3 × 3 grid.

Based on these convergence test results, the Mg_24_(Gd,Y)_5_ structural model was geometrically optimized. The relaxed Mg_24_(Gd,Y)_5_ structure was found to have a lattice parameter of 1.128 nm. This value was found to be in excellent agreement with the 1.126 nm lattice parameter determined by transmission electron microscopy (TEM) in previous studies [29]. Further calculations to examine the site preference of Ag atoms, electronic structure, and mechanical properties were performed using the optimized stable structure as the basis.

## 3. Results and Discussion

### 3.1. Crystal Structure and Stability

Cohesive energy (E_coh_) and formation enthalpy (ΔH) are the two critical thermodynamic parameters that are used to assess the stability and formation capability of alloy phases. Cohesive energy (E_coh_) and formation enthalpy (ΔH) for the Mg_24_(Gd,Y)_5_ phase and its doped structures were calculated using the following equations [30]:

(1) $$E_{cho}=\frac{E_{total}-n_{1}E_{atom}^{Mg}-n_{2}E_{atom}^{Gd}-n_{3}E_{atom}^{Y}-n_{4}E_{atom}^{Ag}}{n_{1}+n_{2}+n_{3}+n_{4}}$$

(2) $$∆H=\frac{E_{total}-n_{1}E_{solid}^{Mg}-n_{2}E_{solid}^{Gd}-n_{3}E_{solid}^{Y}-n_{4}E_{solid}^{Ag}}{n_{1}+n_{2}+n_{3}+n_{4}}$$

Here, E_total_ denotes the total energy of the optimized unit cell. The term $E_{atom}^{i}$ corresponds to the energy of an isolated atom of species i, while $E_{solid}^{i}$ is the energy per atom of the same species i in its stable bulk elemental phase. The symbols n_1_, n_2_, n_3_, and n_4_ denote the numbers of Mg, Gd, Y, and Ag atoms in the unit cell, respectively. Table 2 presents the lattice constants, cohesive energy (E_coh_), and formation energy (ΔH) obtained after geometry optimization for four Ag-doped configurations.

Cohesive energy (E_coh_) is defined as the energy needed to dissociate a crystal into isolated atoms. The lower (and more negative) the Ecoh value, the stronger the interatomic bonding and the more stable the crystal structure [31]. As shown in Table 2, negative cohesive energies were observed for all the alloy models in this study. This result was found to indicate that all doped structures exhibit favorable phase stability. Among all the alloy models, the two Ag-substituted Mg configurations were found to exhibit lower cohesive energies compared to the undoped system. In particular, the Mg_47_Gd_5_Y_5_Ag-2 structure was found to have the lowest cohesive energy (–2.202 eV/atom), which indicates the strongest interatomic bonding. While the cohesive energy provides a measure of phase stability based on isolated atom references, its absolute value can be sensitive to computational details, particularly for spin-polarized species such as Gd. Therefore, formation enthalpy (ΔH) was additionally computed, as it references the energies of bulk elemental phases and is generally more robust for assessing the relative stability between different substitution configurations. This dual approach enables cross-validation of thermodynamic trends and enhances the robustness of our conclusions regarding Ag site preference.

The formation enthalpy (ΔH) is defined as the energy absorbed or released during compound formation and is used to reflect the ease with which alloy phases are formed [30]. A negative value of formation enthalpy is found to indicate that the compound can form spontaneously. The lower (and more negative) the formation enthalpy value, the more readily the alloy phase forms, and vice versa. Specifically, the formation energy of the structure with Ag substituting for Gd was increased (i.e., became less negative) relative to the undoped Mg_24_(Gd,Y)_5_. This indicates that the alloying ability of the doped phase was weakened to some extent. However, the formation enthalpy was slightly decreased for structures with Ag substituting for Mg. In particular, the Mg_47_Gd_5_Y_5_Ag-2 structure, which has four rare earth atoms in its coordination environment, possesses the lowest formation enthalpy (–0.063 eV/atom). This result demonstrates that its alloying ability is the most favorable. This result is consistent with the cohesive energy trend, further implicating that Ag is preferentially incorporated into Mg sites and exhibits greater stability in rare earth-enriched local coordination environments. This finding offers a thermodynamic explanation for a previously reported experimental observation: in fabricated Mg-Gd-Y-Ag-Zr alloys, Ag atoms exhibit a pronounced tendency to segregate within regions enriched with rare-earth elements [32].

In addition, the variation in lattice constants is also worth attention. The substitution of Gd or Y atoms by Ag induced a contraction in the lattice parameter, decreasing from 11.280 Å in the undoped system to approximately 11.237–11.240 Å. This contraction primarily resulted from the significantly smaller atomic size of Ag (1.60 Å) compared to that of Gd (1.80 Å) and Y (1.80 Å). Therefore, the size match of substitutional atoms exerts a significant influence on lattice parameters.

### 3.2. Charge Density

**Figure 4:**
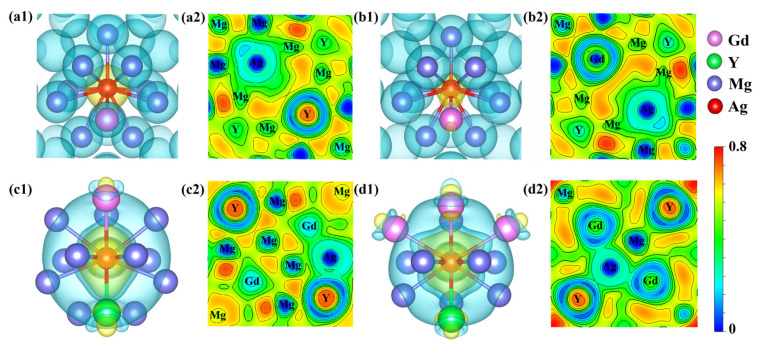
Differential charge density and charge localization density diagrams (**a1**,**a2**) density distribution diagrams of Mg_48_Gd_4_Y_5_Ag; (**b1**,**b2**) density distribution diagrams of Mg_48_Gd_5_Y_4_Ag; (**c1**,**c2**) density distribution diagrams of Mg_47_Gd_5_Y_5_Ag-1; (**d1**,**d2**) density distribution diagrams of Mg_47_Gd_5_Y_5_Ag-2.

In computational materials research, the evolution of electronic structure is critical for understanding microscopic interaction mechanisms. To reveal the effects of Ag doping on the electronic properties and bonding characteristics of Mg_24_(Gd,Y)_5_, the differential charge density (Δρ) and electron localization function (ELF) of the alloy systems were calculated and analyzed. The differential charge density results (Figure 4a1) reveal distinct yellow regions of electron accumulation around Ag atoms and blue-green regions of electron depletion near adjacent Gd and Mg atoms. Electrons are transferred from Gd (x ≈ 1.21) and Mg (x ≈ 1.31), which have lower electronegativity, to Ag (x ≈ 1.93) with higher electronegativity, leading to the formation of an Ag-centered polarized bonding mode with charge polarization toward neighboring atoms. ELF ranges from 0 to 1 and quantifies the degree of electron localization [33]. ELF = 1 denotes perfect electron localization, ELF = 0.5 corresponds to free-electron-gas behavior, and ELF = 0 signifies strong electron delocalization. In the ELF cross-sections (Figure 4a2,d2), Ag–RE and Ag–Mg bonds exhibit ELF values of approximately 0.6 at the bond midpoints, noticeably higher than the value of 0.5 observed for Mg–Mg metallic bonds in the matrix. This elevation indicates enhanced electron localization and stronger bond directionality along these heteroatomic bonds relative to the surrounding metallic matrix.

As depicted in Figure 4c1,d1, distinct “petal-like” electron accumulation regions were formed around Ag atoms after they replaced Mg sites. These electron density accumulations exhibit pronounced spatial directionality and point preferentially toward neighboring rare earth atoms. Meanwhile, mirror-symmetric electron depletion regions were observed near RE atoms, indicating that electrons are predominantly transferred from RE atoms to Ag atoms. By contrast, the electronic interactions between Ag and neighboring Mg atoms are relatively weak. Further comparison of the ELF distributions (Figure 4c2,d2) reveals that the Mg_47_Gd_5_Y_5_Ag-2 alloy exhibits a stronger synergistic interaction between Ag and surrounding RE atoms. The total charge transfer and bonding anisotropy in this structure are significantly higher than those in Mg_47_Gd_5_Y_5_Ag-1. This enhanced interaction facilitates the stable formation of Ag–RE atom clusters or enriched regions. A previous study [24] reported, based on atomic-resolution HAADF-STEM, a distinctive “spine-like periodic segregation structure” composed of alternating Gd-rich and Ag-rich columns at the {10-12} twin boundary (TBII). STEM-EDS line-scan results further confirmed that Ag and Gd exhibit a high degree of elemental distribution synergy, in good agreement with the aforementioned electronic structure analysis.

### 3.3. State Density

**Figure 5:**
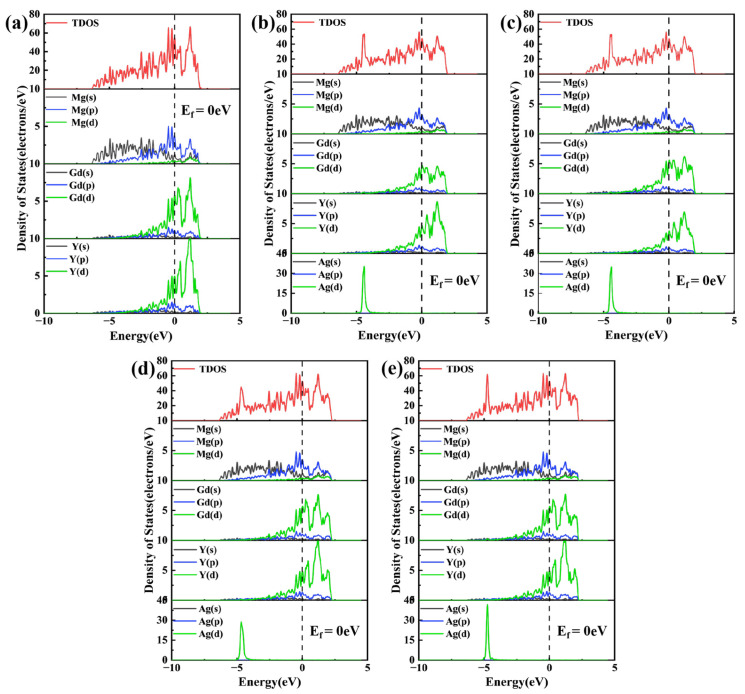
Total density of states (TDOS) and partial density of states (PDOS) (**a**) Mg_24_(Gd,Y)_5_; (**b**) Mg_48_Gd_4_Y_5_Ag; (**c**) Mg_48_Gd_5_Y_4_Ag; (**d**) Mg_47_Gd_5_Y_5_Ag-1; (**e**) Mg_47_Gd_5_Y_5_Ag-2.

To probe the evolution of electronic structure upon Ag doping, the density of states (DOS) for various substitutional configurations of Mg_24_(Gd,Y)_5_ was analyzed, as presented in Figure 5. In these plots, the Fermi level (E_f_) serves as the energy reference, indicated by the vertical dashed line. Regarding the magnetic behavior, both the undoped and Ag-doped Mg_24_(Gd,Y)_5_ phases exhibit weak ferromagnetism, with the magnetic moment primarily originating from the unpaired 4f electrons of Gd atoms. Ag atoms possess a 4d^10^ electronic configuration and make no contribution to magnetism, and their orbital hybridization with rare-earth atoms does not alter the ferromagnetic order.

The total density of states (TDOS) for the Mg_24_(Gd,Y)_5_ phase exhibits distinct features indicative of multi-atom orbital hybridization, as shown in Figure 5a. Near the E_f_, the Mg 3s and 3p states are distributed continuously across a broad energy range. In contrast, the Gd 5d and Y 4d states exhibit sharp, prominent peaks that dominate the overall electronic structure of the phase. At this time, there is a partial overlap region in energy between Mg and rare earths, indicating that there is certain hybridization and bonding interaction, giving the alloy certain metallic bonding and partial covalent characteristics. Following the substitution of Gd atoms by Ag atoms (Figure 4b1,b2), the Ag 4d orbitals exhibited a strong, localized, sharp peak near −5 eV. This distinct peak stems from the 4d^10^5s^1^ electronic configuration of Ag. Hybridization between the Ag 4d and Gd 5d orbitals induced significant changes in the electronic structure. Specifically, the Gd 5d states broadened, and a portion of their density of states shifted to lower energies. These alterations effectively weakened the original bonding interaction between Gd and Mg. When Ag atoms occupy Mg lattice sites (Figure 5d,e), their 4d states exhibit a strong, localized peak near −5 eV. Notably, a broadened, synergistic peak exists in the d orbitals of Ag and adjacent Gd/Y over the −5 eV to Ef range, confirming orbital hybridization within this energy range. Combining the differential charge density analysis in Figure 4d1, it is found that significant charge transfer occurs between Ag and rare earth (RE) atoms in these configurations. This effect is particularly pronounced in the Mg_47_Gd_5_Y_5_Ag-2 structure, which features a rare-earth-enriched local coordination environment and leads to the formation of directional localized bonds. This enhanced electronic interaction, arising from both orbital hybridization and charge redistribution, underpins the superior thermodynamic stability of this configuration, i.e., the lowest formation energy and cohesive energy, as presented in Table 2.

### 3.4. Mechanical Properties

Elastic constants are the key parameters that characterize the linear response of crystalline materials to applied stress. They directly reflect interatomic bonding strength and the material’s macroscopic stiffness. For the cubic crystal system, owing to its high degree of symmetry, elastic behavior is fully described by the three independent elastic constants (C_11_, C_12_, and C_44_) [34]. According to the Born-Huang stability criterion, the mechanical stability of cubic crystals must satisfy the following conditions simultaneously: C_12_ > 0, C_44_ > 0, C_11_ − |C_12_| > 0, and C_11_ + 2C_12_ > 0 [35]. From the C_i__j_ elastic constant results presented in Table 3, it is found that Mg_24_(Gd,Y)_5_ and its four doped alloy structures all meet these stability criteria. This indicates the mechanical stability of these crystal structures.

Based on the calculated elastic constants, the Voigt-Reuss-Hill (VRH) averaging method is employed in this work to systematically evaluate the macroscopic elastic properties of the alloy [36,37]. For all crystal structures, elastic moduli are estimated via the Voigt and Reuss methods, respectively. The Voigt method assumes uniform strain distribution and provides an upper bound for the elastic modulus. The Reuss method assumes uniform stress distribution and yields a lower bound. Their average, the VRH value, is widely accepted as the best estimate of the theoretical polycrystalline elastic modulus. The relevant formulas for calculating elastic constants are given below [35].

(3) $$B_{V}=B_{R}=\frac{1}{3}\left(C_{11}+2C_{12}\right)$$

(4) $$G_{V}=\frac{1}{5}\left(C_{11}-C_{12}+3C_{44}\right)$$

(5) $$G_{R}={\left[\frac{4}{5}{\left(C_{11}-C_{12}\right)}^{-1}+\frac{3}{5}{C_{44}}^{-1}\right]}^{-1}$$

(6) $$B=\frac{1}{2}\left(B_{V}+B_{R}\right)$$

(7) $$G=\frac{1}{2}\left(G_{V}+G_{R}\right)$$

(8) $$E=\frac{9BG}{3B+G}$$

**Figure 6:**
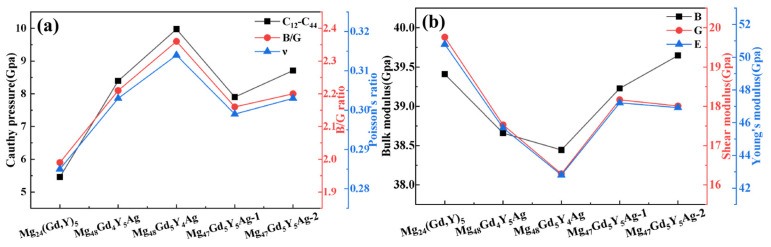
Effects of Ag atom doping on the mechanical properties of Mg_24_(Gd,Y)_5_ (**a**) Bulk modulus, shear modulus, Young’s modulus; (**b**) Cauchy pressure, B/G ratio, Poisson’s ratio ν.

The bulk modulus (B) is the key parameter that characterizes a material’s resistance to uniform compression in the elastic regime. A larger B value indicates that the material is more difficult to compress. The shear modulus (G) and Young’s modulus (E) are closely related to material hardness. Although the relationship between elastic moduli and hardness differs among materials, larger E and G values generally correspond to higher material hardness [38]. As presented in Figure 6a, the bulk modulus, shear modulus, and Young’s modulus of Mg_24_(Gd,Y)_5_ are relatively high. This indicates excellent compression resistance and high macroscopic hardness in this alloy phase. Upon Ag doping, the moduli of Mg_48_Gd_4_Y_5_Ag and Mg_48_Gd_5_Y_4_Ag decrease significantly. This reduction is mainly ascribed to lattice distortion and the weakening of local atomic bonding (especially Ag–Mg bonds) induced by Ag atoms. These effects weaken the alloy’s overall resistance to volumetric compression and shear deformation. In contrast, the moduli of Mg_48_Gd_5_Y_5_Ag-1 and Mg_48_Gd_5_Y_5_Ag-2 show a rebound. For Mg_48_Gd_5_Y_5_Ag-2 in particular, its bulk modulus approaches that of the undoped alloy. Its shear modulus and Young’s modulus also increase significantly. This observation indicates that the interaction between Ag atoms and the surrounding atoms (especially rare earth atoms) is effectively optimized in these doping configurations. This optimization enhances the alloy’s resistance to deformation and thus improves its macroscopic mechanical properties.

In metallic materials research, the Cauchy pressure (C_12_ − C_44_) is an important indicator for evaluating plasticity. A larger Cauchy pressure usually corresponds to better material plasticity [34]. The ratio of bulk modulus to shear modulus (B/G) can be used to assess material toughness based on the Pugh criterion: a larger B/G ratio indicates greater toughness; when B/G > 1.75, the material exhibits ductility, and conversely, it shows brittleness [39]. Additionally, Poisson’s ratio (ν) serves as another criterion: when ν > 0.26, the material displays toughness [40]. Figure 6b shows that the undoped Mg_24_(Gd,Y)_5_ exhibits relatively low values for the Cauchy pressure, the B/G ratio, and Poisson’s ratio. Upon Ag doping, these three parameters exhibit an increasing trend, indicating significant improvements in the plasticity and toughness of the alloy phase. This change also reveals that while the material’s plasticity and toughness are enhanced, its strength and hardness may decrease. This suggests a trade-off between strength and plasticity-toughness during performance optimization.

However, it should be noted that these parameters primarily reflect the elastic response of the Mg_24_(Gd,Y)_5_ phase itself and do not directly translate to the macroscopic tensile ductility of multiphase alloys. The overall ductility of the alloy is also significantly influenced by microstructural features, including precipitate-free zones (PFZs), the distribution and morphology of second phases, grain size, and crystallographic texture. First, the addition of Ag promotes the formation of precipitate-free zones (PFZs) along the grain boundaries [41]. During plastic deformation, PFZs at the grain boundaries alleviate stress concentration. These zones facilitate dislocation slip and twinning, thereby enhancing the alloy’s ductility. For example, in a study on the Mg-2.4Gd-0.4Ag-0.1Zr alloy, rough regions are observed on its fracture surface in addition to smooth grain boundary areas. These regions are closely associated with local plastic deformation at the grain-boundary PFZs, indicating a positive role of PFZs in improving the alloy’s plasticity and toughness. This finding is consistent with the result that Ag doping enhances the material’s plasticity and toughness. Additionally, the grain refinement strengthening mechanism also contributes significantly to the improved plasticity and toughness of the alloy. The grain boundaries effectively hinder dislocation motion and disperse stress. These effects suppress crack initiation and propagation. Bao et al. [42] reported in a study on Mg-10.5Gd-2Y-0.3Zr and Mg-10.5Gd-2Y-1.5Ag-0.3Zr alloys that Ag addition refines the average grain size from 101 μm to 60 μm. This grain refinement is primarily attributed to the formation of Ag-Gd intermetallic compounds. These compounds exhibit a specific crystallographic orientation relationship with the α-Mg matrix, allowing them to serve as effective heterogeneous nucleation sites. By promoting nucleation and suppressing subsequent grain growth, they increase the total grain boundary area. This microstructural refinement, in turn, contributes to the alloy’s enhanced overall mechanical properties.

## 4. Experimental Verification

To verify the reliability of the aforementioned first-principles calculations and clarify the regulation mechanisms of Ag doping on the microstructure and mechanical properties of Mg-Gd-Y-Zr alloys, Ag-doped Mg-Gd-Y-Zr alloys are prepared experimentally in this section. X-ray diffraction (XRD), transmission electron microscopy (TEM), and room-temperature tensile tests are employed for systematic characterization in terms of phase composition, microstructural morphology, and mechanical response. This enables cross-validation between the theoretical calculations and the experimental results.

### 4.1. Sample Printing and WAAM Process

In this work, VW63K (measured composition: Mg-6.54Gd-3.93Y-0.41Zr, wt.%) and VW63K-Ag (measured composition: Mg-7.12Gd-2.26Y-0.38Zr-0.94Ag, wt.%) alloy wires with a diameter of 1.6 mm were employed as feedstock. Samples were fabricated via single-pass multi-layer deposition using the CMT-WAAM process. The specific deposition parameters were as follows: a current of 110 A, a voltage of 12 V, a wire feed speed of 10 m/min, a welding speed of 9 m/min, a stick-out of 0.012 m, and an interlayer dwell time of 60 s to mitigate interlayer heat accumulation. Ar was used as the shielding gas at a flow rate of 20 L/min during deposition. This prevented oxidation and contamination of the Mg alloy at elevated temperatures.

The phase composition of the samples was analyzed using an X-ray diffractometer (XRD, D8 ADVANCE, Bruker AXS, Karlsruhe, Germany). The test conditions were as follows: Cu Kα radiation, operating voltage 40 kV, scanning range 20–80°, step size 0.02°, and scanning speed 2°/min. Microstructural characterization was performed using a transmission electron microscope (TEM, ThermoFisher Talos F200X, FEI company, Hillsboro, OR, USA). TEM specimens were first mechanically ground and punched into discs. They were then thinned using a GATAN PIPS II(Gatan, Inc., Pleasanton, CA, USA) ion mill at ±5° with 4 keV accelerating voltage to produce electron-transparent regions. Tensile properties were measured using a SUNS UTM2000 (Shenzhen SUNS Technology Stock Co., Ltd., Shenzhen, China) universal testing machine per the ASTM E8/E8M standard [43]. A crosshead speed of 1 mm/min was employed, with at least 3 parallel specimens tested per group, and the average value was reported.

### 4.2. Microstructural Characterization

**Figure 7:**
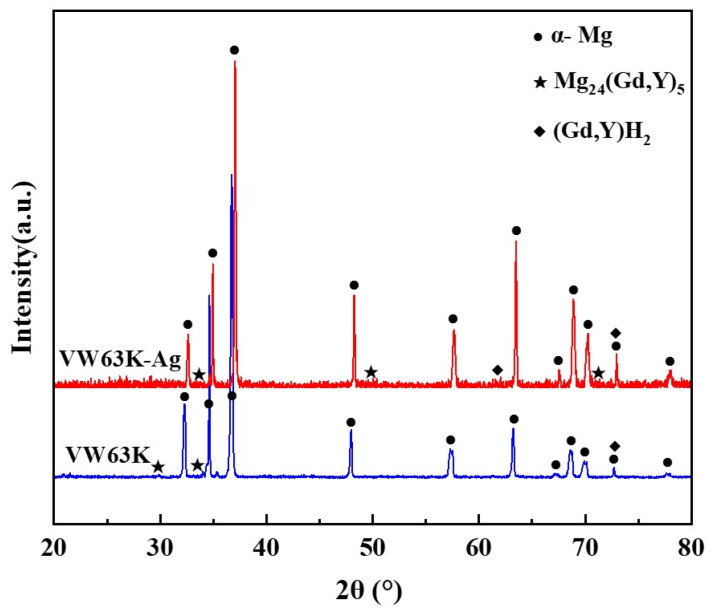
XRD diffraction patterns of the two alloys.

The XRD phase analysis in Figure 7 reveals diffraction peaks of the α-Mg matrix and Mg_24_Y_5_ (PDF#31-0817) in both VW63K and VW63K-Ag alloys. Gd and Y exhibit similar atomic structure, chemical behavior, and solid solution characteristics in the Mg alloy system. During the formation of Mg_24_Y_5_, some Gd atoms substitute for Y sites. This phase is thus more accurately denoted as Mg_24_(Gd,Y)_5_ [44]. Additionally, a trace amount of the cubic (Gd, Y)H_2_ phase is detected in the XRD pattern. Hydrogen exhibits a high diffusion rate (on the order of 10^−11^ m^2^/s) in Mg. Trace hydrogen remaining in the alloy during WAAM or environmental exposure reacts with enriched Gd/Y to form this (Gd, Y)H_2_ intermetallic compound [45]. The cubic phase is present in minimal quantities. Its effect on the mechanical properties of both alloys is negligible and thus is not discussed further. Comparison of the XRD patterns for the two alloys reveals that no new Ag-based phases are detected in the VW63K-Ag alloy. This indicates that Ag atoms exist in solid solution within the Mg_24_(Gd,Y)_5_ phase or the α-Mg matrix.

Notably, the diffraction peaks of the Mg_24_(Gd,Y)_5_ phase in the VW63K-Ag alloy shift slightly to higher 2θ angles relative to the undoped VW63K alloy. This peak shift confirms lattice contraction of the Mg_24_(Gd,Y)_5_ phase upon Ag doping, which is consistent with the reduced lattice parameters reported in Table 2. Although the atomic radii of Ag and Mg are similar, the enhanced orbital hybridization between Ag and RE atoms (discussed in Section 3.3) optimizes interatomic bonding, resulting in a more compact lattice structure that ultimately leads to lattice contraction.

**Figure 8:**
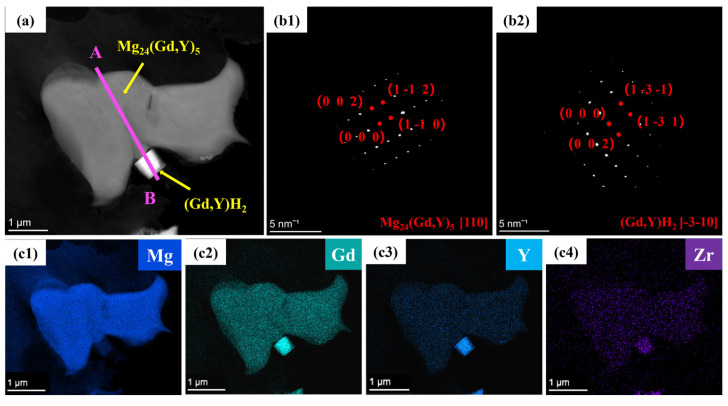
TEM images of the VW63K sample (**a**) Bright-field image; (**b1**,**b2**) SAED patterns of Mg_24_(Gd,Y)_5_ and (Gd, Y)H_2_; (**c1**–**c4**) HAADF-STEM mapping.

To further clarify the microstructural characteristics of the two alloys, transmission electron microscopy (TEM) was conducted on the alloys. Results from high-angle annular dark-field scanning transmission electron microscopy (HAADF-STEM) and elemental mapping (Figure 8a,c1–c4) reveal that the Mg_24_(Gd,Y)_5_ second phase is distributed as islands within the α-Mg matrix. Gd and Y are significantly enriched in this second phase, whereas Zr exhibits a homogeneous distribution. Diffraction spots in Figure 8b1 confirm that this phase exhibits a body-centered cubic (BCC) structure with a lattice parameter of 1.12 nm, in agreement with reported values [46,47]. Additionally, a cubic phase 800 nm in size is observed in Figure 8a. Elemental mapping reveals that this phase is enriched in Gd and Y. The selected area electron diffraction (SAED) pattern in Figure 8b2 further verifies a face-centered cubic (FCC) structure for this phase, consistent with the (Gd, Y)H_2_ phase.

**Figure 9:**
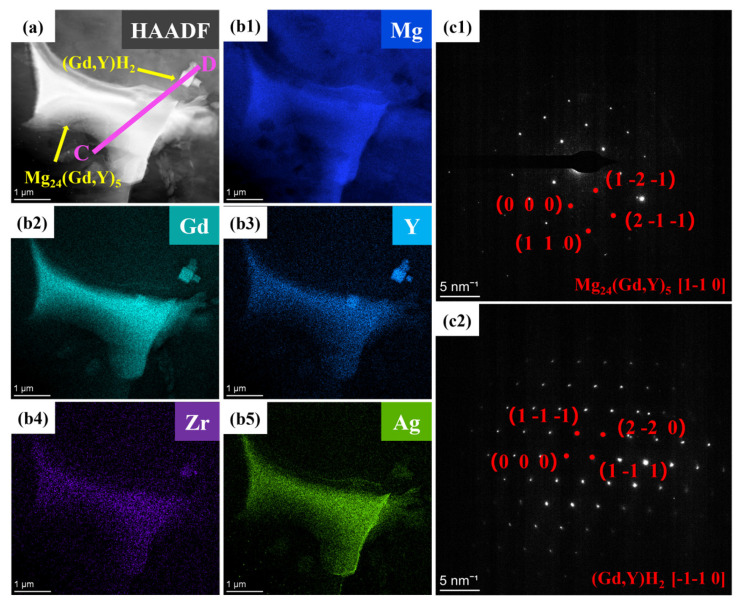
TEM images of the VW63K-Ag sample (**a**) Bright-field image; (**b1**–**b5**) HAADF-STEM mapping; (**c1**,**c2**) Mg_24_(Gd,Y)_5_ and (Gd,Y)H_2_.

**Figure 10:**
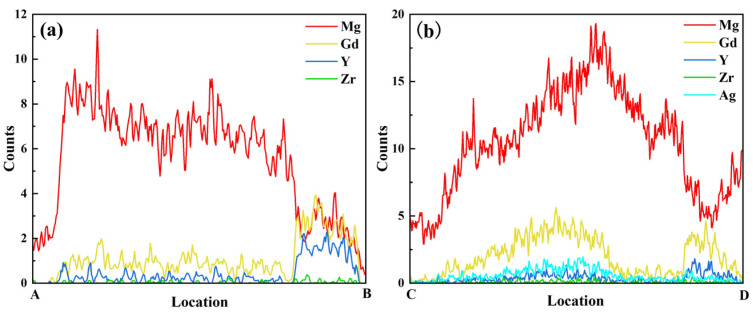
Elements distribution maps by line scanning (**a**) line AB in Figure 8; (**b**) line CD in Figure 9.

Elemental mapping for Ag (Figure 9b5) reveals that Ag atoms are predominantly distributed within the Mg_24_(Gd,Y)_5_ phase. They exhibit significant segregation in the rare-earth-enriched regions. The SAED patterns in Figure 9c1,c2 show that diffraction spots along the corresponding zone axes for the Mg_24_(Gd,Y)_5_ and (Gd,Y)H_2_ phases are regularly arranged. This further confirms the structural integrity of these two types of phases. Additionally, line scans along lines AB and CD (Figure 10) show more clearly that the Ag distribution signal closely matches the Gd and Y distribution trends. This distribution behavior agrees well with the first-principles prediction that Ag segregates in the rare-earth-enriched regions of the Mg_24_(Gd,Y)_5_ phase. This observation further validates the reliability of the simulations.

### 4.3. Mechanical Properties

**Figure 11:**
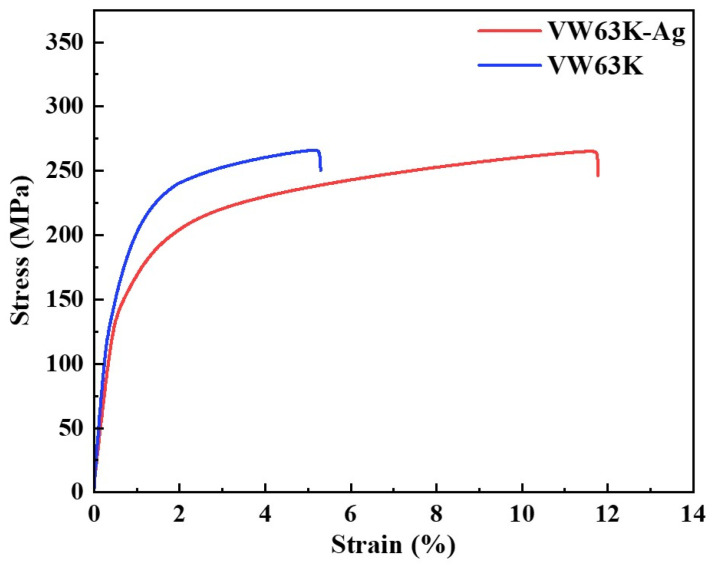
Stress–strain curves of the two alloys.

Figure 11 presents the room-temperature tensile stress–strain curves, which clearly reveal the modulating effect of Ag doping on the mechanical properties of the VW63K alloy. The VW63K alloy (blue curve) exhibits high yield strength (YS) and ultimate tensile strength (UTS), but low elongation to fracture. In contrast, the VW63K-Ag alloy (red curve) shows a slightly lower yield strength. Its fracture strain is markedly enhanced to above 12%, demonstrating a synergistic effect of moderately reduced strength and significantly improved plasticity.

It is important to note that there are differences in Gd and Y contents between the two alloys. These compositional variations may affect the chemical composition of the second phase, precipitate volume fraction, and local solute distribution, thereby influencing the overall mechanical behavior. For instance, a higher Gd content may promote the formation of more rare-earth-enriched precipitates, enhancing the precipitation strengthening effect; in contrast, a lower Y content may slightly alter the tensile strength and elongation of the alloy [48,49]. Nevertheless, the primary focus of this study remains on elucidating the role of Ag in regulating the phase stability and mechanical properties of Mg_24_(Gd,Y)_5_, which is supported by first-principles calculations. The undoped Mg_24_(Gd,Y)_5_ phase exhibits high bulk, shear, and Young’s moduli. These are consistent with the high intrinsic strength of the VW63K alloy. However, its Cauchy pressure, B/G ratio, and Poisson’s ratio are relatively low. This indicates limited plastic deformability of the phase, in agreement with the observed poor plasticity of the VW63K alloy. Following Ag doping, the elastic moduli of these configurations exhibit some fluctuations. However, their Cauchy pressure, B/G ratio, and Poisson’s ratio all show an increasing trend. This indicates that the enhanced plasticity of the Mg_24_(Gd,Y)_5_ phase in the VW63K-Ag alloy effectively suppresses the brittle fracture propensity during deformation. The alloy thus achieves greater plastic deformability with a moderate reduction in yield strength. Meanwhile, the variations in elastic modulus correlate with the macroscopic strength tuning across atomic-to-macroscopic length scales. This result validates the first-principles prediction of a strength-plasticity trade-off induced by Ag doping. It establishes a closed-loop validation between the underlying microscopic mechanisms and the macroscopic mechanical behavior.

## 5. Conclusions

This study employs a combined approach of first-principles calculations and experimental characterization to systematically investigate the site preference, electronic structure, and mechanical properties of Ag atoms in the Mg_24_(Gd,Y)_5_ phase. Microstructure and phase composition of the Mg-Gd-Y-Zr alloy fabricated via WAAM were analyzed. This work enables cross-validation between theoretical simulations and experimental results. The main conclusions are as follows:

(1) Ag atoms preferentially occupy Mg sites in the Mg_24_(Gd,Y)_5_ phase and exhibit significant segregation in the rare-earth-enriched regions. This behavior is also validated experimentally by microstructural characterization.
(2) In all substitutionally doped configurations, Ag atoms behave as electron acceptors and form chemical bonds with mixed covalent–ionic character with neighboring rare-earth (RE) atoms. The strengths of orbital hybridization and interatomic interactions are strongly modulated by the substitution site and local coordination environment.
(3) The undoped Mg_24_(Gd,Y)_5_ phase possesses high intrinsic mechanical properties. In the VW63K-Ag alloy, the enhanced plasticity of the Mg_24_(Gd,Y)_5_ phase effectively suppresses brittle fracture propensity during deformation. This enables the alloy to achieve greater plastic deformability with a moderate reduction in yield strength, leading to an optimized strength–plasticity synergy.

## Figures and Tables

**Table 1:** Comparison of the local coordination environments for the two crystallographically equivalent 24g Mg sites in Mg_24_(Gd,Y)_5_.

	Mg1	Mg2
Wyckoff (relaxed)	(0.14323, 0.53018, 0.85677)	(0.08943, 0.27978, 0.91058)
Wyckoff (this work)	(0.14335, 0.53212, 0.85650)	(0.08981, 0.28071, 0.91015)
Coordination Number	13	12
Coordination Composition	11 Mg + 2 RE	8 Mg + 4 RE
Average Bond Length	3.27 Å	3.21 Å

**Table 2:** Lattice constants, cohesive energies (E_cho_), and formation energies (ΔH) of Mg_24_(Gd,Y)_5_ and its four designed Ag-doped structures.

	Lattice Constants/Å	E_cho_/eV	ΔH/eV
Mg_24_(Gd,Y)_5_	a = 11.280	−2.177	−0.055
Mg_48_Gd_4_Y_5_Ag	a = 11.237	−2.140	−0.047
Mg_48_Gd_5_Y_4_Ag	a = 11.240	−2.108	−0.047
Mg_47_Gd_5_Y_5_Ag-1	a = 11.247	−2.198	−0.058
Mg_47_Gd_5_Y_5_Ag-2	a = 11.255	−2.202	−0.063

**Table 3:** Elastic constants (C_ij_/GPa); Cauchy pressure (C_12_-C_44_/GPa); bulk modulus (B/GPa); shear modulus (G/GPa); Young’s modulus (E/GPa); B/G ratio (B/G); and Poisson’s ratio (ν) of Mg_24_(Gd,Y)_5_ and its doped structures.

	C_11_	C_12_	C_44_	C_12_-C_44_	B	G	E	B/G	ν
Mg_24_(Gd,Y)_5_	75.079	21.574	16.107	5.457	39.409	19.760	50.792	1.99	0.285
Mg_48_Gd_4_Y_5_Ag	71.127	22.425	14.031	8.394	38.659	17.527	45.679	2.21	0.303
Mg_48_Gd_5_Y_4_Ag	70.314	22.511	12.536	9.975	38.445	16.281	42.802	2.36	0.314
Mg_47_Gd_5_Y_5_Ag-1	72.672	22.506	14.606	7.900	39.228	18.166	47.210	2.16	0.299
Mg_47_Gd_5_Y_55_Ag-2	72.173	23.383	14.673	8.710	39.646	18.009	46.922	2.20	0.303

## Data Availability

The original contributions presented in this study are included in the article. Further inquiries can be directed to the corresponding authors.

## Abbreviations

The following abbreviations are used in this manuscript:

CMT-WAAM	Cold Metal Transfer Wire Arc Additive Manufacturing
UTS	Ultimate tensile strength
AM	Additive manufacturing
YS	Yield strength
DFT	Density functional theory
BCC	Body-centered cubic
VASP	Vienna ab initio Simulation Package
PBE	Perdew-Burke-Ernzerhof
GGV	Generalized gradient approximation
PAW	Projector augmented wave
DOS	Density of states
PBC	Periodic boundary conditions
TEM	Transmission electron microscopy
ELF	Electron localization function
RE	Rare earth
PFZs	Precipitate-free zones
VRH	Voigt-Reuss-Hill
XRD	X-ray diffraction
HAADF-STE	High-angle annular dark-field scanning transmission electron microscopy
SAED	Selected area electron diffraction
FCC	Face-centered cubic
